# Supplementary material for: Maternal ambient air pollution exposure and risk of stillbirth in Georgia, USA
Source: Environ Epidemiol. 2026 Jul 7;10(4):e505. doi: 10.1097/EE9.0000000000000505 (PMC13344931; doi:10.1097/EE9.0000000000000505)

Supplementary Material

Table S1. Pearson Correlation Coefficients matrix between weekly levels of all ten pollutants from 2005 to 2014.

	CO	NO3	NOx	O3	PM10	PM2.5	EC	OC	NO3	NH3
CO	1	0.812	0.786	-0.028	0.397	0.509	0.744	0.551	0.433	0.481
NO2	0.812	1	0.888	-0.01	0.314	0.407	0.762	0.358	0.47	0.358
NOx	0.786	0.888	1	-0.211	0.184	0.273	0.752	0.29	0.48	0.211
O3	-0.028	-0.01	-0.211	1	0.589	0.56	0.082	0.245	-0.352	0.393
PM10	0.397	0.314	0.184	0.589	1	0.817	0.444	0.527	-0.039	0.645
PM2.5	0.509	0.407	0.273	0.56	0.817	1	0.542	0.671	0.148	0.798
EC	0.744	0.762	0.752	0.082	0.444	0.542	1	0.531	0.363	0.384
OC	0.551	0.358	0.29	0.245	0.527	0.671	0.531	1	0.3	0.604
NO3	0.433	0.47	0.48	-0.352	-0.039	0.148	0.363	0.3	1	0.315
NH4	0.481	0.358	0.211	0.393	0.645	0.798	0.384	0.604	0.315	1

Table S2. Pearson Correlation Coefficients between different exposure windows within each of ten air pollutants.

Pollutants	Cor (T1, T2)	Cor (T1, T3)	Cor (T2, T3)
CO	0.9414	-0.0008	-0.0019
NO2	0.9166	-0.0007	-0.0031
NOx	0.8034	0.0006	0.0005
O3	0.0647	-0.0034	-0.0028
PM10	0.4445	0.0022	-0.0012
PM2.5	0.631	0.0022	0.0033
EC	0.8264	0.0047	0.0064
OC	0.8841	0.0034	0.0042
NO3	0.3709	-0.0016	-0.0081
NH4	0.8104	0.0022	0.0045

We also constructed the same table for stillbirths with gestational age >=30 weeks. Because many stillbirths have a short third trimester, correlations between third trimester exposure and earlier trimesters can seem low. Restricting to later stillbirths reduces can give a clearer depiction of trimester correlations.

Pollutants	Cor (T1, T2)	Cor (T1, T3)	Cor (T2, T3)
CO	0.9407	0.8974	0.9421
NO2	0.9157	0.8572	0.9247
NOx	0.7995	0.6422	0.8128
O3	0.0337	-0.6803	0.1081
PM10	0.431	-0.0064	0.4763
PM2.5	0.6257	0.3857	0.6451
EC	0.8266	0.7208	0.8375
OC	0.8857	0.8307	0.8868
NO3	0.3544	-0.1123	0.4154
NH4	0.8082	0.6802	0.8206

Table S3. Hazard ratio (HR) estimates per unit IQR increase and 95% CI's for all ten pollutants during five exposure windows (the first month, weekly exposure, the first trimester exposure, the second trimester exposure, and the third trimester exposure) for all stillbirths, stillbirths that happened in the second trimester, and stillbirths that happened in the third trimester. Estimates are adjusted for individual-level maternal age, marital status, race & ethnicity, conception year, and tract-level median household income.

Exposure time window	Pollutants	All stillbirth (n=8384)	2nd trimester (n=4670)	3rd trimester (n=3714)
The first month	CO	1.018 (0.957, 1.082)	1.074 (0.974, 1.185)	0.938 (0.852, 1.032)
	NO2	0.973 (0.938, 1.009)	1.019 (0.954, 1.088)	0.908 (0.845, 0.977)
	NOx	0.985 (0.955, 1.017)	0.996 (0.952, 1.041)	0.966 (0.920, 1.015)
	O3	0.959 (0.909, 1.012)	0.956 (0.873, 1.048)	0.961 (0.862, 1.072)
	PM10	0.993 (0.966, 1.020)	1.033 (0.976, 1.092)	0.935 (0.879, 0.993)
	PM2.5	0.991 (0.964, 1.018)	1.023 (0.965, 1.085)	0.948 (0.890, 1.010)
	EC	0.978 (0.948, 1.009)	1.012 (0.961, 1.066)	0.927 (0.854, 1.006)
	OC	1.006 (0.960, 1.055)	1.031 (0.959, 1.109)	0.977 (0.892, 1.070)
	NO3	1.004 (0.972, 1.037)	1.020 (0.969, 1.074)	0.991 (0.921, 1.066)
	NH4	1.003 (0.969, 1.037)	1.053 (0.972, 1.140)	0.939 (0.872, 1.010)
Weekly	CO	1.077 (1.023, 1.133)	1.152 (1.053, 1.261)	0.980 (0.913, 1.052)
	NO2	1.038 (0.997, 1.081)	1.112 (1.041, 1.188)	0.946 (0.870, 1.029)
	NOx	1.024 (0.995, 1.053)	1.072 (1.019, 1.128)	0.959 (0.914, 1.007)
	O3	1.059 (1.001, 1.122)	1.075 (0.983, 1.176)	1.049 (0.987, 1.114)
	PM10	1.040 (1.000, 1.081)	1.075 (1.029, 1.123)	0.999 (0.936, 1.068)
	PM2.5	1.016 (0.976, 1.058)	1.056 (1.001, 1.114)	0.973 (0.919, 1.031)
	EC	1.028 (0.982, 1.077)	1.069 (1.003, 1.140)	0.985 (0.917, 1.057)
	OC	1.019 (0.966, 1.074)	1.043 (0.980, 1.111)	0.987 (0.919, 1.060)
	NO3	1.012 (0.978, 1.047)	1.046 (0.997, 1.098)	0.975 (0.923, 1.030)
	NH4	1.022 (0.982, 1.064)	1.060 (1.007, 1.115)	0.985 (0.927, 1.047)
The first trimester cumulative average	CO	1.060 (0.981, 1.144)	1.105 (0.979, 1.247)	0.998 (0.891, 1.118)
	NO2	1.002 (0.947, 1.059)	1.050 (0.958, 1.151)	0.939 (0.861, 1.025)
	NOx	1.002 (0.953, 1.053)	1.024 (0.956, 1.097)	0.965 (0.893, 1.042)
	O3	0.974 (0.924, 1.026)	0.937 (0.842, 1.042)	1.030 (0.905, 1.173)
	PM10	1.000 (0.959, 1.043)	1.039 (0.951, 1.136)	0.943 (0.851, 1.045)
	PM2.5	1.002 (0.975, 1.030)	1.037 (0.970, 1.110)	0.954 (0.879, 1.036)
	EC	0.993 (0.937, 1.052)	1.026 (0.932, 1.130)	0.948 (0.868, 1.035)
	OC	1.037 (0.984, 1.094)	1.035 (0.940, 1.141)	1.043 (0.936, 1.162)
	NO3	1.008 (0.972, 1.045)	1.069 (1.004, 1.139)	0.934 (0.857, 1.018)
	NH4	1.013 (0.984, 1.043)	1.072 (0.986, 1.166)	0.940 (0.857, 1.031)
The second trimester cumulative average	CO	1.079 (0.976, 1.193)	1.126 (0.993, 1.276)	0.979 (0.819, 1.170)
	NO2	1.064 (1.002, 1.131)	1.121 (1.033, 1.215)	0.963 (0.845, 1.097)
	NOx	1.026 (0.983, 1.070)	1.084 (0.998, 1.178)	0.917 (0.791, 1.064)
	O3	1.049 (0.972, 1.131)	1.062 (0.975, 1.156)	1.035 (0.921, 1.163)
	PM10	1.073 (1.041, 1.106)	1.089 (1.045, 1.136)	1.033 (0.977, 1.091)
	PM2.5	1.045 (1.006, 1.085)	1.067 (1.011, 1.125)	0.997 (0.933, 1.066)
	EC	1.045 (0.997, 1.096)	1.078 (1.001, 1.160)	0.987 (0.891, 1.093)
	OC	1.024 (0.967, 1.083)	1.090 (1.011, 1.175)	0.909 (0.813, 1.017)
	NO3	1.016 (0.962, 1.072)	1.089 (1.015, 1.169)	0.890 (0.803, 0.985)
	NH4	1.058 (1.022, 1.095)	1.065 (1.016, 1.116)	1.041 (0.956, 1.134)
The third trimester cumulative average	CO	-	-	1.010 (0.880, 1.159)
	NO2	-	-	1.002 (0.872, 1.152)
	NOx	-	-	0.958 (0.886, 1.035)
	O3	-	-	1.122 (1.012, 1.244)
	PM10	-	-	1.000 (0.956, 1.045)
	PM2.5	-	-	0.988 (0.924, 1.055)
	EC	-	-	0.996 (0.913, 1.087)
	OC	-	-	0.961 (0.850, 1.087)
	NO3	-	-	0.971 (0.898, 1.049)
	NH4	-	-	0.963 (0.870, 1.066)

Table S4. Hazard ratio (HR) estimates per unit IQR increase and 95% CI's for all ten pollutants during five exposure windows (the first month, weekly exposure, the first trimester exposure, the second trimester exposure, and the third trimester exposure) for stillbirths that happened after the 37th week of pregnancy. Estimates are adjusted for individual-level maternal age, marital status, race & ethnicity, conception year, and tract-level median household income.

Exposure time window	Pollutants	Stillbirth after the 37th week
The first month	CO	0.860 (0.682, 1.085)
	NO2	0.879 (0.702, 1.100)
	NOx	0.949 (0.794, 1.134)
	O3	0.962 (0.814, 1.138)
	PM10	0.930 (0.811, 1.066)
	PM2.5	0.953 (0.850, 1.068)
	EC	0.831 (0.647, 1.067)
	OC	0.942 (0.777, 1.143)
	NO3	0.934 (0.828, 1.054)
	NH4	0.998 (0.873, 1.141)
Weekly	CO	1.068 (0.896, 1.273)
	NO2	1.029 (0.839, 1.261)
	NOx	1.028 (0.937, 1.129)
	O3	1.122 (0.947, 1.328)
	PM10	1.019 (0.917, 1.133)
	PM2.5	1.042 (0.931, 1.167)
	EC	1.032 (0.939, 1.134)
	OC	0.972 (0.863, 1.094)
	NO3	0.977 (0.880, 1.085)
	NH4	0.988 (0.892, 1.095)
The first trimester cumulative average	CO	0.933 (0.731, 1.190)
	NO2	0.942 (0.744, 1.192)
	NOx	1.005 (0.859, 1.175)
	O3	1.060 (0.813, 1.383)
	PM10	1.146 (0.874, 1.503)
	PM2.5	1.087 (0.874, 1.353)
	EC	0.884 (0.712, 1.099)
	OC	0.938 (0.703, 1.253)
	NO3	0.776 (0.643, 0.936)
	NH4	1.034 (0.773, 1.383)
The second trimester cumulative average	CO	0.838 (0.513, 1.369)
	NO2	1.552 (1.193, 2.019)
	NOx	1.104 (0.894, 1.362)
	O3	1.026 (0.739, 1.424)
	PM10	1.062 (0.858, 1.313)
	PM2.5	0.966 (0.799, 1.168)
	EC	1.118 (0.925, 1.350)
	OC	0.800 (0.547, 1.172)
	NO3	0.867 (0.694, 1.082)
	NH4	1.011 (0.840, 1.218)
The third trimester cumulative average	CO	0.728 (0.373, 1.422)
	NO2	0.767 (0.471, 1.250)
	NOx	0.756 (0.511, 1.118)
	O3	1.349 (0.815, 2.232)
	PM10	1.135 (0.975, 1.321)
	PM2.5	1.055 (0.850, 1.309)
	EC	0.977 (0.668, 1.429)
	OC	0.889 (0.629, 1.256)
	NO3	1.209 (0.946, 1.545)
	NH4	1.127 (0.872, 1.457)

Table S5. Hazard ratio (HR) estimates per unit IQR increase and 95% CI's for all ten pollutants during five exposure windows (the first month, weekly exposure, the first trimester exposure, the second trimester exposure, and the third trimester exposure) for all stillbirths, stillbirths that happened in the second trimester, and stillbirths that happened in the third trimester, adjusting for weekly average maximum temperature. Estimates are adjusted for individual-level maternal age, marital status, race & ethnicity, conception year, and tract-level median household income.

Exposure time window	Pollutants	All stillbirth (n=8384)	2nd trimester (n=4670)	3rd trimester (n=3714)
The first month	CO	1.011 (0.952, 1.074)	1.066 (0.968, 1.175)	0.933 (0.850, 1.024)
	NO2	0.974 (0.938, 1.010)	1.020 (0.954, 1.091)	0.909 (0.846, 0.977)
	NOx	0.985 (0.955, 1.016)	0.995 (0.952, 1.040)	0.966 (0.920, 1.015)
	O3	0.941 (0.888, 0.997)	0.934 (0.844, 1.033)	0.948 (0.854, 1.052)
	PM10	0.980 (0.952, 1.009)	1.019 (0.966, 1.076)	0.923 (0.865, 0.984)
	PM2.5	0.982 (0.953, 1.011)	1.013 (0.951, 1.078)	0.941 (0.884, 1.000)
	EC	0.970 (0.942, 1.000)	1.003 (0.951, 1.057)	0.921 (0.850, 0.997)
	OC	0.997 (0.949, 1.047)	1.020 (0.944, 1.102)	0.970 (0.884, 1.064)
	NO3	1.019 (0.986, 1.052)	1.041 (0.981, 1.104)	1.000 (0.927, 1.079)
	NH4	1.004 (0.970, 1.038)	1.054 (0.973, 1.142)	0.940 (0.873, 1.011)
Weekly	CO	1.076 (1.024, 1.131)	1.152 (1.054, 1.260)	0.978 (0.912, 1.050)
	NO2	1.039 (0.998, 1.081)	1.112 (1.042, 1.188)	0.947 (0.872, 1.028)
	NOx	1.024 (0.995, 1.054)	1.072 (1.019, 1.128)	0.959 (0.914, 1.007)
	O3	1.059 (0.999, 1.122)	1.076 (0.983, 1.178)	1.046 (0.981, 1.115)
	PM10	1.039 (0.996, 1.084)	1.076 (1.028, 1.127)	0.997 (0.929, 1.069)
	PM2.5	1.016 (0.974, 1.058)	1.056 (1.001, 1.115)	0.972 (0.916, 1.031)
	EC	1.028 (0.980, 1.078)	1.069 (1.002, 1.141)	0.983 (0.914, 1.058)
	OC	1.018 (0.964, 1.075)	1.043 (0.980, 1.111)	0.986 (0.917, 1.060)
	NO3	1.016 (0.982, 1.050)	1.050 (1.003, 1.100)	0.979 (0.922, 1.040)
	NH4	1.023 (0.982, 1.065)	1.060 (1.007, 1.116)	0.987 (0.929, 1.048)
The first trimester cumulative average	CO	1.058 (0.980, 1.142)	1.103 (0.978, 1.245)	0.995 (0.889, 1.114)
	NO2	1.004 (0.949, 1.063)	1.052 (0.958, 1.157)	0.942 (0.864, 1.028)
	NOx	1.003 (0.954, 1.055)	1.026 (0.956, 1.100)	0.967 (0.895, 1.046)
	O3	0.962 (0.911, 1.016)	0.926 (0.830, 1.033)	1.017 (0.897, 1.153)
	PM10	0.992 (0.953, 1.033)	1.036 (0.952, 1.127)	0.930 (0.838, 1.032)
	PM2.5	0.997 (0.971, 1.025)	1.035 (0.968, 1.105)	0.947 (0.875, 1.024)
	EC	0.990 (0.934, 1.050)	1.024 (0.930, 1.128)	0.944 (0.862, 1.033)
	OC	1.033 (0.976, 1.092)	1.032 (0.932, 1.142)	1.037 (0.927, 1.159)
	NO3	1.022 (0.986, 1.059)	1.088 (1.020, 1.161)	0.944 (0.861, 1.034)
	NH4	1.014 (0.985, 1.045)	1.073 (0.986, 1.168)	0.942 (0.857, 1.035)
The second trimester cumulative average	CO	1.077 (0.975, 1.189)	1.126 (0.995, 1.274)	0.975 (0.816, 1.166)
	NO2	1.064 (1.001, 1.131)	1.120 (1.033, 1.215)	0.963 (0.845, 1.098)
	NOx	1.026 (0.983, 1.070)	1.084 (0.998, 1.178)	0.918 (0.794, 1.062)
	O3	1.047 (0.969, 1.133)	1.063 (0.973, 1.162)	1.030 (0.918, 1.157)
	PM10	1.074 (1.039, 1.109)	1.092 (1.046, 1.141)	1.030 (0.972, 1.091)
	PM2.5	1.045 (1.006, 1.085)	1.068 (1.011, 1.128)	0.994 (0.932, 1.060)
	EC	1.044 (0.995, 1.096)	1.079 (1.002, 1.161)	0.984 (0.882, 1.097)
	OC	1.022 (0.967, 1.081)	1.091 (1.012, 1.177)	0.907 (0.812, 1.013)
	NO3	1.019 (0.966, 1.075)	1.093 (1.011, 1.182)	0.892 (0.806, 0.988)
	NH4	1.058 (1.022, 1.095)	1.065 (1.016, 1.116)	1.040 (0.956, 1.132)
The third trimester cumulative average	CO	-	-	1.006 (0.875, 1.156)
	NO2	-	-	1.003 (0.873, 1.152)
	NOx	-	-	0.958 (0.887, 1.035)
	O3	-	-	1.119 (1.007, 1.243)
	PM10	-	-	0.996 (0.948, 1.046)
	PM2.5	-	-	0.984 (0.921, 1.051)
	EC	-	-	0.993 (0.907, 1.087)
	OC	-	-	0.959 (0.846, 1.087)
	NO3	-	-	0.977 (0.896, 1.066)
	NH4	-	-	0.963 (0.870, 1.066)

Table S6. Hazard ratio (HR) estimates per unit IQR increase and 95% CI's for all ten pollutants during five exposure windows (the first month, weekly exposure, the first trimester exposure, the second trimester exposure, and the third trimester exposure) for all stillbirths, stillbirths that happened in the second trimester, and stillbirths that happened in the third trimester, adjusting for weekly average minimum temperature. Estimates are adjusted for individual-level maternal age, marital status, race & ethnicity, conception year, and tract-level median household income.

Exposure time window	Pollutants	All stillbirth (n=8384)	2nd trimester (n=4670)	3rd trimester (n=3714)
The first month	CO	1.018 (0.957, 1.082)	1.074 (0.974, 1.185)	0.938 (0.852, 1.032)
	NO2	0.973 (0.938, 1.009)	1.019 (0.954, 1.088)	0.908 (0.845, 0.977)
	NOx	0.985 (0.955, 1.017)	0.996 (0.952, 1.041)	0.966 (0.920, 1.015)
	O3	0.959 (0.909, 1.012)	0.956 (0.873, 1.048)	0.961 (0.862, 1.072)
	PM10	0.993 (0.966, 1.020)	1.033 (0.976, 1.092)	0.935 (0.879, 0.993)
	PM2.5	0.991 (0.964, 1.018)	1.023 (0.965, 1.085)	0.948 (0.890, 1.010)
	EC	0.978 (0.948, 1.009)	1.012 (0.961, 1.066)	0.927 (0.854, 1.006)
	OC	1.006 (0.960, 1.055)	1.031 (0.959, 1.109)	0.977 (0.892, 1.070)
	NO3	1.004 (0.972, 1.037)	1.020 (0.969, 1.074)	0.991 (0.921, 1.066)
	NH4	1.003 (0.969, 1.037)	1.053 (0.972, 1.140)	0.939 (0.872, 1.010)
Weekly	CO	1.078 (1.022, 1.136)	1.159 (1.058, 1.269)	0.974 (0.907, 1.046)
	NO2	1.040 (0.996, 1.085)	1.125 (1.052, 1.202)	0.935 (0.861, 1.016)
	NOx	1.025 (0.995, 1.056)	1.080 (1.026, 1.137)	0.952 (0.908, 0.998)
	O3	1.060 (1.000, 1.123)	1.079 (0.985, 1.182)	1.046 (0.982, 1.113)
	PM10	1.040 (1.000, 1.081)	1.076 (1.030, 1.124)	0.998 (0.933, 1.066)
	PM2.5	1.017 (0.976, 1.059)	1.061 (1.005, 1.119)	0.969 (0.913, 1.027)
	EC	1.029 (0.982, 1.079)	1.076 (1.010, 1.147)	0.979 (0.911, 1.052)
	OC	1.019 (0.965, 1.076)	1.048 (0.984, 1.117)	0.981 (0.915, 1.052)
	NO3	1.013 (0.980, 1.047)	1.059 (1.008, 1.112)	0.965 (0.909, 1.024)
	NH4	1.022 (0.981, 1.065)	1.063 (1.009, 1.121)	0.982 (0.922, 1.045)
The first trimester cumulative average	CO	1.059 (0.981, 1.144)	1.104 (0.978, 1.247)	0.998 (0.891, 1.118)
	NO2	0.999 (0.941, 1.060)	1.046 (0.948, 1.155)	0.938 (0.857, 1.027)
	NOx	0.999 (0.945, 1.057)	1.021 (0.945, 1.104)	0.964 (0.891, 1.044)
	O3	0.975 (0.925, 1.029)	0.939 (0.843, 1.045)	1.031 (0.907, 1.171)
	PM10	1.004 (0.966, 1.043)	1.047 (0.965, 1.137)	0.941 (0.850, 1.041)
	PM2.5	1.004 (0.977, 1.031)	1.040 (0.973, 1.111)	0.954 (0.880, 1.034)
	EC	0.993 (0.938, 1.052)	1.027 (0.933, 1.130)	0.948 (0.867, 1.036)
	OC	1.039 (0.984, 1.097)	1.037 (0.940, 1.145)	1.043 (0.936, 1.163)
	NO3	1.000 (0.959, 1.043)	1.065 (0.991, 1.144)	0.925 (0.843, 1.014)
	NH4	1.012 (0.980, 1.045)	1.071 (0.983, 1.167)	0.940 (0.855, 1.033)
The second trimester cumulative average	CO	1.079 (0.976, 1.193)	1.126 (0.993, 1.276)	0.979 (0.819, 1.169)
	NO2	1.068 (1.005, 1.135)	1.128 (1.040, 1.224)	0.963 (0.842, 1.102)
	NOx	1.028 (0.985, 1.072)	1.089 (1.003, 1.182)	0.917 (0.794, 1.060)
	O3	1.048 (0.972, 1.130)	1.061 (0.975, 1.156)	1.035 (0.922, 1.162)
	PM10	1.073 (1.041, 1.106)	1.088 (1.043, 1.135)	1.033 (0.977, 1.092)
	PM2.5	1.045 (1.006, 1.085)	1.066 (1.011, 1.125)	0.997 (0.934, 1.065)
	EC	1.045 (0.997, 1.097)	1.078 (1.002, 1.160)	0.987 (0.890, 1.094)
	OC	1.024 (0.967, 1.084)	1.091 (1.012, 1.176)	0.909 (0.813, 1.017)
	NO3	1.019 (0.967, 1.074)	1.100 (1.014, 1.193)	0.888 (0.800, 0.985)
	NH4	1.059 (1.022, 1.096)	1.066 (1.017, 1.117)	1.041 (0.956, 1.134)
The third trimester cumulative average	CO	-	-	1.010 (0.880, 1.160)
	NO2	-	-	0.993 (0.863, 1.142)
	NOx	-	-	0.953 (0.881, 1.031)
	O3	-	-	1.123 (1.013, 1.245)
	PM10	-	-	1.002 (0.958, 1.049)
	PM2.5	-	-	0.989 (0.925, 1.056)
	EC	-	-	0.995 (0.912, 1.087)
	OC	-	-	0.960 (0.850, 1.085)
	NO3	-	-	0.962 (0.887, 1.043)
	NH4	-	-	0.962 (0.868, 1.065)

Table S7. Hazard ratios (HRs) and 95% CIs from single-pollutant and two-pollutant Cox proportional hazards models. Two pollutant models included NO_2 adjusted for $PM_{2.5}$, O_3 adjusted for $PM_{2.5}$, and $PM_{2.5}$ adjusted for O_3.

Exposure time window	Pollutants	All stillbirth (n=8384)	2nd trimester (4670)	3rd trimester (3714)
The first month	NO2	0.973 (0.938, 1.009)	1.019 (0.954, 1.088)	0.908 (0.845, 0.977)
	NO2 + PM2.5	0.975 (0.934, 1.017)	1.009 (0.942, 1.081)	0.926 (0.863, 0.993)
	O3	0.959 (0.909, 1.012)	0.956 (0.873, 1.048)	0.961 (0.862, 1.072)
	O3 + PM2.5	0.953 (0.888, 1.022)	0.915 (0.822, 1.019)	1.004 (0.884, 1.141)
	PM2.5	0.991 (0.964, 1.018)	1.023 (0.965, 1.085)	0.948 (0.890, 1.010)
	PM2.5 + O3	1.009 (0.971, 1.047)	1.057 (0.987, 1.132)	0.947 (0.880, 1.018)
Weekly	NO2	1.038 (0.997, 1.081)	1.112 (1.041, 1.188)	0.946 (0.870, 1.029)
	NO2 + PM2.5	1.033 (0.992, 1.075)	1.089 (1.013, 1.170)	0.956 (0.887, 1.030)
	O3	1.059 (1.001, 1.122)	1.075 (0.983, 1.176)	1.049 (0.987, 1.114)
	O3 + PM2.5	1.069 (1.005, 1.137)	1.044 (0.947, 1.151)	1.103 (1.017, 1.197)
	PM2.5	1.016 (0.976, 1.058)	1.056 (1.001, 1.114)	0.973 (0.919, 1.031)
	PM2.5 + O3	0.989 (0.947, 1.033)	1.038 (0.983, 1.096)	0.935 (0.868, 1.007)
The first trimester cumulative average	NO2	1.002 (0.947, 1.059)	1.050 (0.958, 1.151)	0.939 (0.861, 1.025)
	NO2 + PM2.5	1.001 (0.944, 1.061)	1.040 (0.948, 1.139)	0.952 (0.876, 1.035)
	O3	0.974 (0.924, 1.026)	0.937 (0.842, 1.042)	1.030 (0.905, 1.173)
	O3 + PM2.5	0.964 (0.897, 1.035)	0.888 (0.792, 0.996)	1.086 (0.932, 1.266)
	PM2.5	1.002 (0.975, 1.030)	1.037 (0.970, 1.110)	0.954 (0.879, 1.036)
	PM2.5 + O3	1.015 (0.974, 1.057)	1.079 (1.004, 1.159)	0.928 (0.841, 1.024)
The second trimester cumulative average	NO2	1.064 (1.002, 1.131)	1.121 (1.033, 1.215)	0.963 (0.845, 1.097)
	NO2 + PM2.5	1.038 (0.975, 1.105)	1.081 (0.978, 1.195)	0.958 (0.830, 1.106)
	O3	1.049 (0.972, 1.131)	1.062 (0.975, 1.156)	1.035 (0.921, 1.163)
	O3 + PM2.5	1.020 (0.936, 1.113)	1.017 (0.922, 1.122)	1.040 (0.909, 1.190)
	PM2.5	1.045 (1.006, 1.085)	1.067 (1.011, 1.125)	0.997 (0.933, 1.066)
	PM2.5 + O3	1.035 (0.993, 1.078)	1.054 (0.993, 1.119)	0.987 (0.912, 1.069)
The third trimester cumulative average	NO2	-	-	1.002 (0.872, 1.152)
	NO2 + PM2.5	-	-	1.005 (0.859, 1.177)
	O3	-	-	1.122 (1.012, 1.244)
	O3 + PM2.5	-	-	1.174 (1.012, 1.362)
	PM2.5	-	-	0.988 (0.924, 1.055)
	PM2.5 + O3	-	-	0.932 (0.848, 1.025)

Table S8. Hazard ratio (HR) estimates per unit IQR increase and 95% CIs for all ten pollutants for stillbirths occurring between the 37th to the 42nd gestational week (full term) and stillbirths occurring before the 37th gestational week.

Exposure time window	Pollutants	37 to 42 weeks	Less than 37 weeks
The first month	CO	0.851 (0.709, 1.021)	1.030 (0.948, 1.118)
	NO2	0.825 (0.719, 0.945)	0.999 (0.919, 1.086)
	NOx	0.932 (0.849, 1.024)	1.000 (0.939, 1.065)
	O3	0.959 (0.841, 1.093)	1.004 (0.914, 1.104)
	PM10	0.927 (0.847, 1.014)	1.028 (0.974, 1.084)
	PM2.5	0.936 (0.852, 1.029)	0.992 (0.933, 1.055)
	EC	0.842 (0.709, 1.000)	1.016 (0.955, 1.080)
	OC	0.911 (0.762, 1.090)	0.996 (0.932, 1.064)
	NO3	0.850 (0.752, 0.961)	1.049 (0.983, 1.118)
	NH4	0.962 (0.859, 1.078)	1.055 (0.982, 1.134)
Weekly	CO	1.014 (0.855, 1.203)	1.051 (0.990, 1.115)
	NO2	1.028 (0.871, 1.213)	1.004 (0.914, 1.103)
	NOx	1.038 (0.966, 1.116)	1.013 (0.953, 1.078)
	O3	1.092 (0.990, 1.203)	1.057 (0.971, 1.150)
	PM10	0.995 (0.907, 1.091)	1.036 (0.995, 1.079)
	PM2.5	1.012 (0.925, 1.108)	1.013 (0.960, 1.069)
	EC	1.032 (0.936, 1.137)	1.021 (0.953, 1.093)
	OC	0.946 (0.854, 1.048)	1.038 (0.980, 1.099)
	NO3	1.039 (0.966, 1.117)	1.025 (0.976, 1.077)
	NH4	0.977 (0.873, 1.093)	1.011 (0.961, 1.063)
The first trimester cumulative average	CO	0.878 (0.708, 1.089)	1.024 (0.907, 1.157)
	NO2	0.868 (0.740, 1.018)	1.036 (0.969, 1.107)
	NOx	0.941 (0.834, 1.061)	1.010 (0.946, 1.078)
	O3	1.042 (0.885, 1.228)	1.003 (0.877, 1.147)
	PM10	1.056 (0.847, 1.317)	1.088 (1.023, 1.157)
	PM2.5	1.036 (0.867, 1.238)	1.029 (0.967, 1.095)
	EC	0.885 (0.753, 1.041)	1.028 (0.961, 1.100)
	OC	0.906 (0.699, 1.175)	1.065 (0.969, 1.171)
	NO3	0.740 (0.630, 0.868)	1.091 (0.984, 1.211)
	NH4	0.993 (0.801, 1.231)	1.078 (1.002, 1.160)
The second trimester cumulative average	CO	0.830 (0.584, 1.180)	1.088 (0.948, 1.248)
	NO2	1.276 (0.971, 1.676)	1.001 (0.911, 1.100)
	NOx	1.094 (0.893, 1.341)	1.013 (0.953, 1.077)
	O3	1.042 (0.834, 1.303)	1.088 (0.999, 1.184)
	PM10	1.098 (0.933, 1.292)	1.076 (1.016, 1.140)
	PM2.5	1.001 (0.874, 1.146)	1.070 (1.001, 1.143)
	EC	1.017 (0.846, 1.222)	1.046 (0.939, 1.164)
	OC	0.770 (0.575, 1.032)	1.082 (1.008, 1.162)
	NO3	0.914 (0.758, 1.104)	1.050 (1.010, 1.092)
	NH4	1.011 (0.855, 1.196)	1.066 (0.990, 1.149)
The third trimester cumulative average	CO	0.899 (0.560, 1.444)	1.031 (0.828, 1.284)
	NO2	0.855 (0.601, 1.217)	1.030 (0.833, 1.274)
	NOx	0.928 (0.716, 1.203)	1.047 (0.877, 1.250)
	O3	1.127 (0.799, 1.589)	1.099 (0.926, 1.306)
	PM10	1.057 (0.893, 1.251)	0.926 (0.851, 1.007)
	PM2.5	1.028 (0.810, 1.305)	0.904 (0.816, 1.002)
	EC	1.012 (0.712, 1.436)	0.956 (0.850, 1.077)
	OC	0.819 (0.658, 1.019)	1.007 (0.847, 1.198)
	NO3	1.223 (1.029, 1.453)	0.904 (0.802, 1.018)
	NH4	1.146 (0.911, 1.442)	0.787 (0.686, 0.903)

Figure S1. Hazard ratio (HR) estimates per unit IQR increase and 95% CIs for all ten pollutants during all five exposure windows restricted to stillbirths occurring after the 37th week of pregnancy.

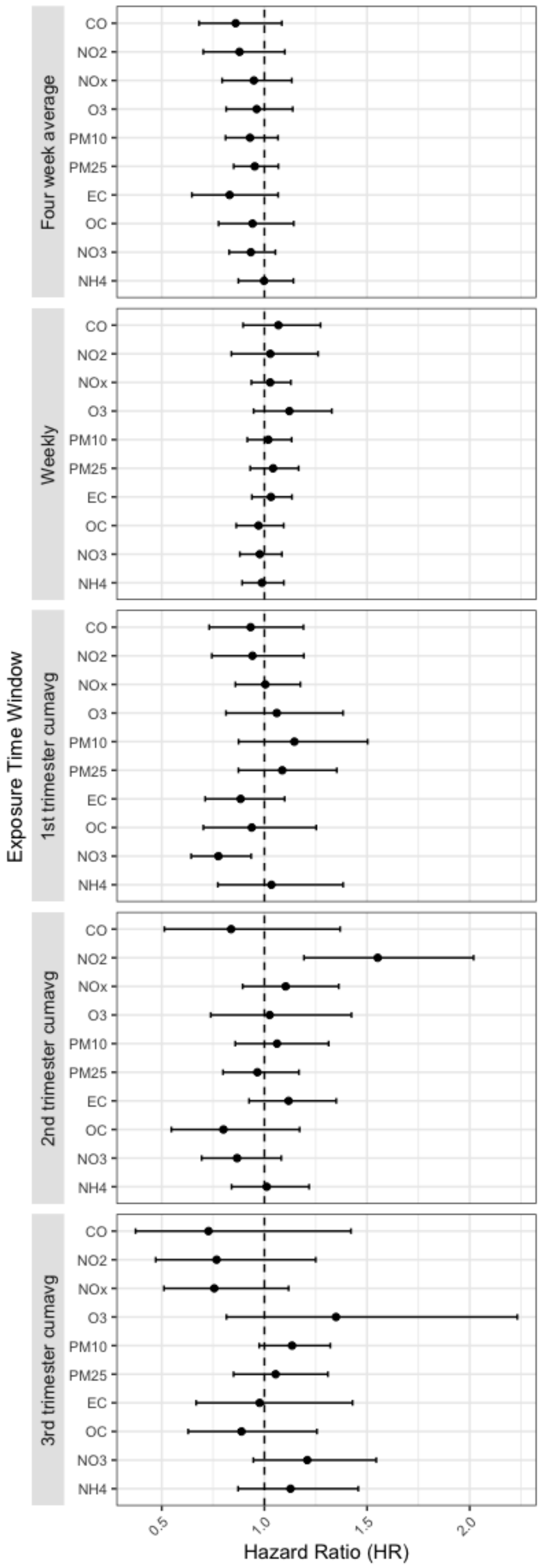

Figure S2. Comparison of hazard ratio (HR) estimates per unit IQR increase and 95% CIs for all ten pollutants during all exposure windows (the first week, weekly the first trimester, the second trimester, and the third trimester exposure) for all stillbirths, stillbirths that happened in the second trimester, and stillbirths that happened in the third trimester between main analysis and sensitivity analysis adjusting for weekly average maximum temperature.

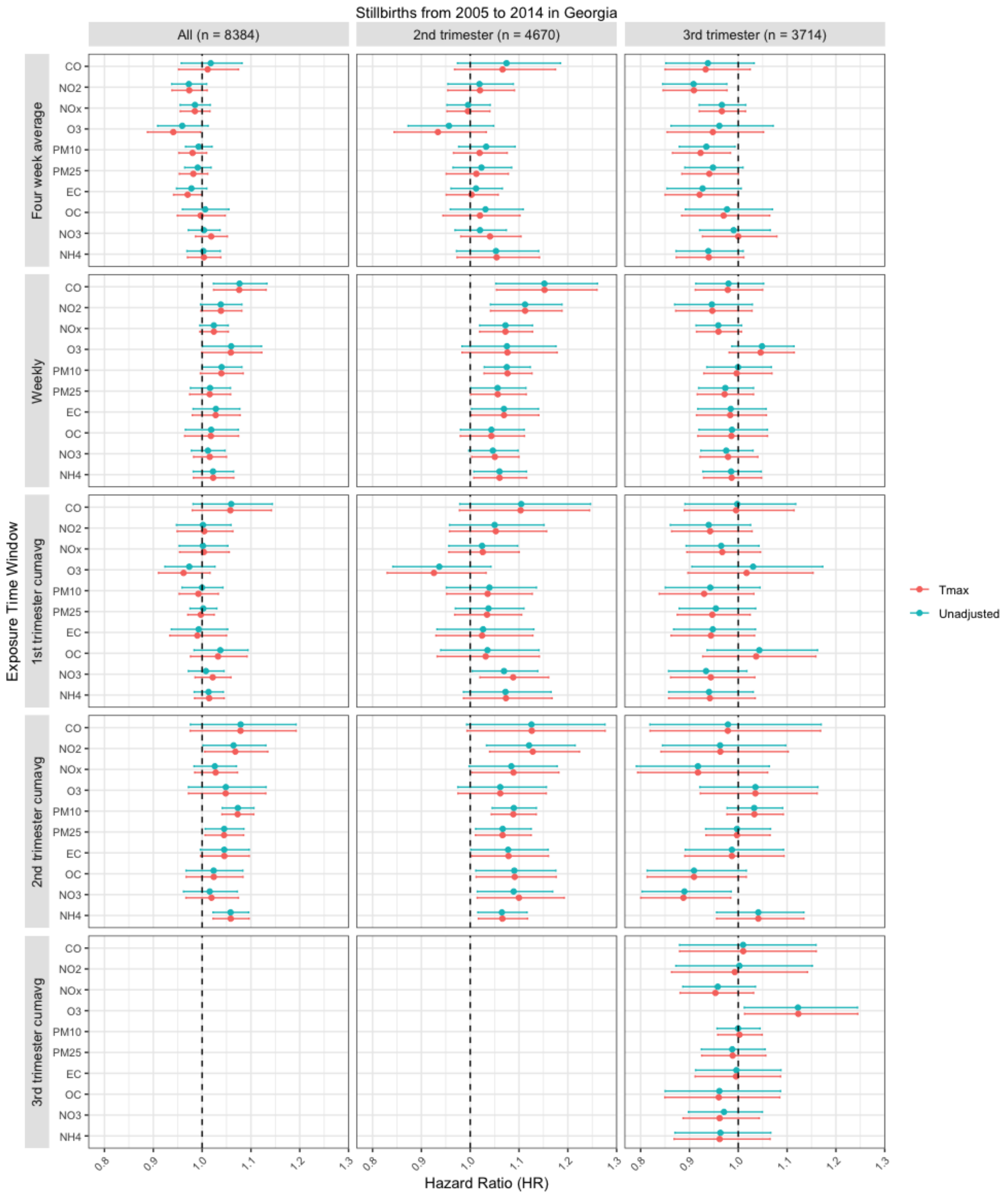

Figure S3. Comparison of hazard ratio (HR) estimates per unit IQR increase and 95% CI's for all ten pollutants during all exposure windows (the first week, weekly the first trimester, the second trimester, and the third trimester exposure) for all stillbirths, stillbirths that happened in the second trimester, and stillbirths that happened in the third trimester between main analysis and sensitivity analysis adjusting for weekly average minimum temperature.

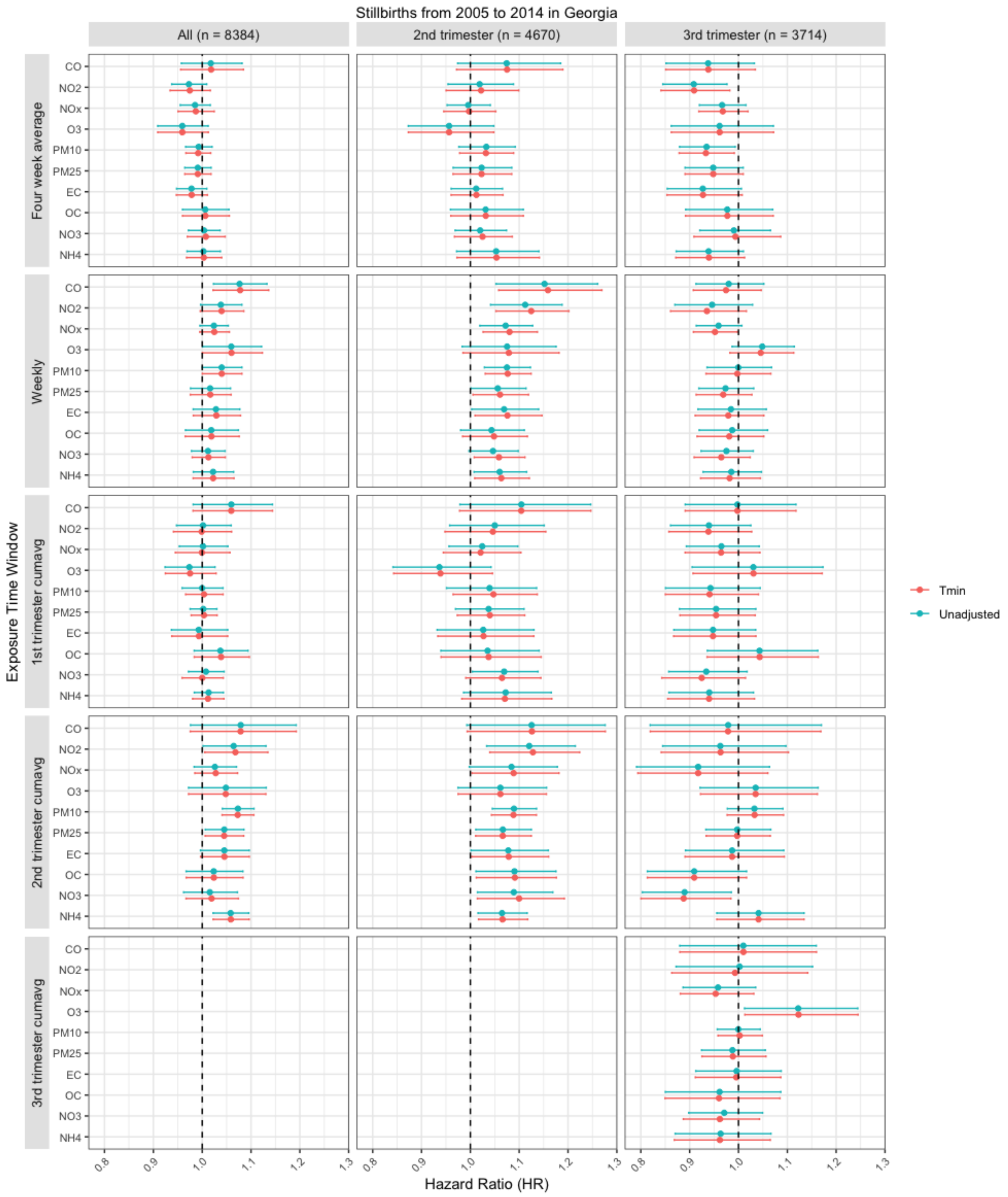

Figure S4. Comparison of hazard ratio (HR) estimates per unit IQR increase and 95% CI's for single-pollutant and two-pollutant model estimates for NO_2, O_3, and $PM_{2.5}$. Hazard ratios (HRs) and 95% confidence intervals correspond to an interquartile range increase in pollutant exposure. Two-pollutant models adjusted NO_2 for $PM_{2.5}$, O_3 for $PM_{2.5}$, and $PM_{2.5}$ for O_3.

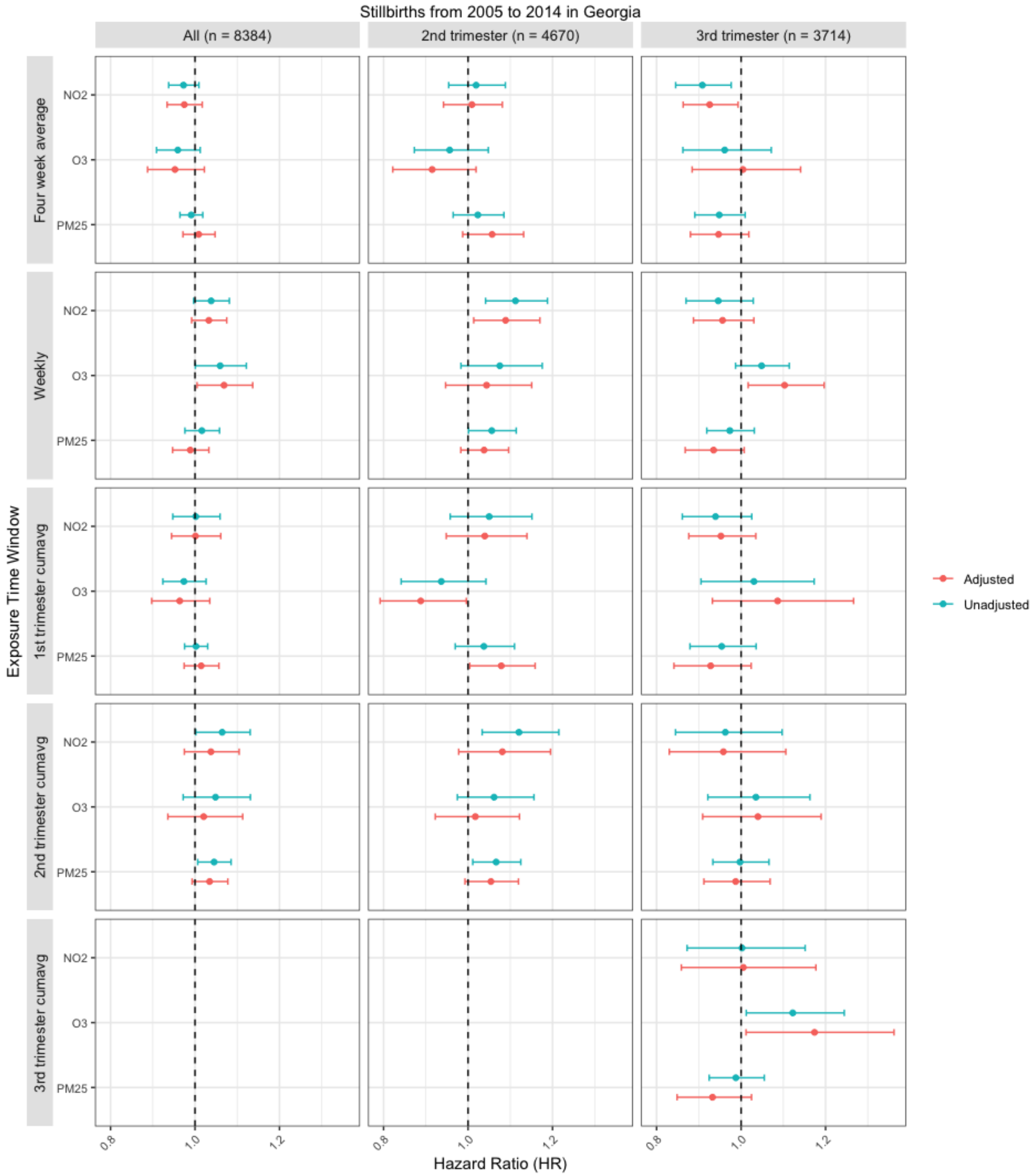

Figure S5. Comparison of hazard ratio (HR) estimates per unit IQR increase and 95% CIs for all ten pollutants during all exposure windows between stillbirths that happened from the 37th to the 42nd gestational week (full term) and stillbirths occurring before the 37th gestational week.

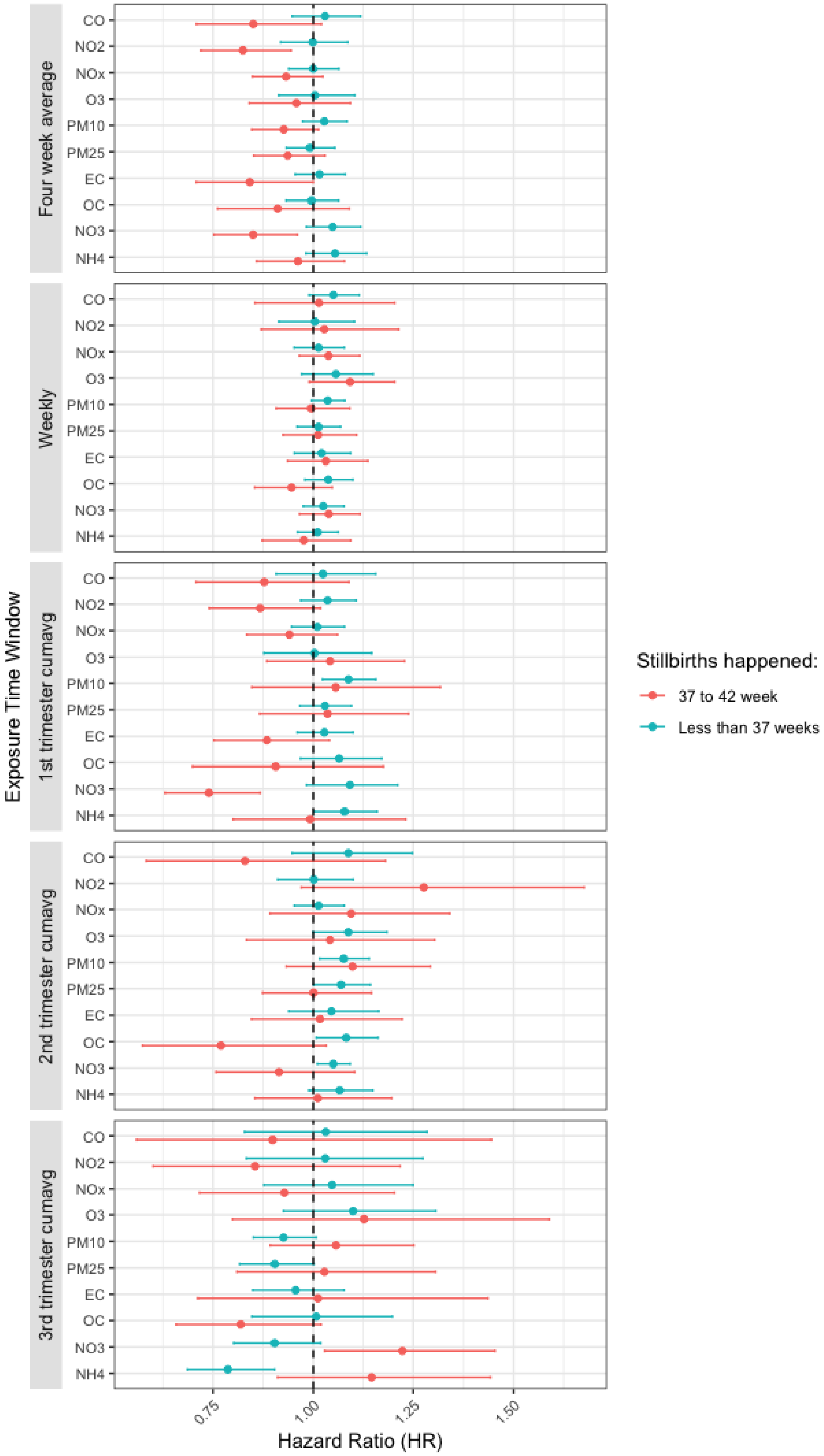

Supplement: Supplementary file 1 [file ee9-10-e505-s001.pdf]
